# Visualising Berry phase and diabolical points in a quantum exciton-polariton billiard

**DOI:** 10.1038/srep37653

**Published:** 2016-11-25

**Authors:** E. Estrecho, T. Gao, S. Brodbeck, M. Kamp, C. Schneider, S. Höfling, A. G. Truscott, E. A. Ostrovskaya

**Affiliations:** 1 Nonlinear Physics Centre, Research School of Physics and Engineering, The Australian National University, Canberra, ACT 2601, Australia; 2 Technische Physik and Wilhelm-Conrad-Röntgen Research Center for Complex Material Systems, Universität Würzburg, Am Hubland, D-97074 Würzburg, Germany; 3 SUPA, School of Physics and Astronomy, University of St Andrews, St Andrews KY16 9SS, UK; 4 Laser Physics Centre, Research School of Physics and Engineering, The Australian National University, Canberra, ACT 2601, Australia

## Abstract

Diabolical points (spectral degeneracies) can naturally occur in spectra of two-dimensional quantum systems and classical wave resonators due to simple symmetries. Geometric Berry phase is associated with these spectral degeneracies. Here, we demonstrate a diabolical point and the corresponding Berry phase in the spectrum of hybrid light-matter quasiparticles—exciton-polaritons in semiconductor microcavities. It is well known that sufficiently strong optical pumping can drive exciton-polaritons to quantum degeneracy, whereby they form a macroscopically populated quantum coherent state similar to a Bose-Einstein condensate. By pumping a microcavity with a spatially structured light beam, we create a two-dimensional quantum billiard for the exciton-polariton condensate and demonstrate a diabolical point in the spectrum of the billiard eigenstates. The fully reconfigurable geometry of the potential walls controlled by the optical pump enables a striking experimental visualization of the Berry phase associated with the diabolical point. The Berry phase is observed and measured by direct imaging of the macroscopic exciton-polariton probability densities.

Geometric Berry phase in wave systems is a phenomenon of accumulation of phase of the energy eigenstates during cyclic adiabatic evolution^1^. In Hermitian quantum systems, the geometric connection of the states of a continuously evolving system with time-varying parameters coincides with the connection of stationary eigenstates in the space of the system’s parameters^2^. The Berry phase is therefore a direct consequence of topological properties of the system’s eigenvalue surfaces in the parameter space. Since its discovery, Berry phase has been shown to arise in a wide range of physical systems, where it is associated with measurable physical effects. The notable examples are molecular dynamics^3^, polarization rotation and the optical spin-Hall effect^4^,^5^,^6^, as well as orbital magnetism and Hall effects of electronic states in solid-state systems^7^,^8^,^9^. Moreover, it was speculated that geometric phases in quantum systems could be useful for information processing because of intrinsic topological protection^10^,^11^.

The most simple and intuitive form of geometric phase in a quantum wave system is associated with spectral degeneracies, i.e. conical intersections of energy surfaces of two eigenstates. Such diabolical points in the space of parameters can be naturally engineered in quantum billiards—two-dimensional hard-wall resonators for quantum waves, in which spectral degeneracies occur in a two-parameter space^12^,^13^. In the simplest case of a two-level degeneracy, the eigenstates of the billiard accumulate a geometric Berry phase of *π* when a diabolical point is encircled in the space of two parameters responsible for the system’s shape. The geometric phase accumulation manifests itself in the rotation of nodal lines of the corresponding wavefunctions^12^,^13^. This geometric phase has been observed in macroscopic classical microwave resonators^14^, and nanoscale electron quantum corrals^10^,^11^.

In this work, we visualise the geometric phase of energy eigenstates of a macroscopic quantum system composed of microcavity exciton-polaritons, which bridges the gap between macroscopic classical and nanoscopic quantum waves. Exciton-polaritons are bosonic quasiparticles arising via hybridisation of strongly coupled excitons and photons in a semiconductor microcavity^15^. Driven by an optical pump, they undergo bosonic condensation into a macroscopically occupied quantum coherent state^16^,^17^,^18^,^19^,^20^,^21^, whose energy and probability density can be directly imaged via microcavity photoluminescence. Thus, the exciton-polariton condensate is a macroscopic quantum system that spans a range of regimes between classical and quantum waves, while lending itself to optical manipulation and observation *in-situ*.

In order to visualise the Berry phase, we construct a two-level system with a diabolical point in the spectrum by confining condensed exciton-polaritons in a quantum billiard—an optically-defined resonator for macroscopic quantum waves^22^. The walls of the resonator are induced by a spatially structured optical pump with the energy tuned far above the energy of the condensing exciton-polaritons. The pump therefore creates a spatially localised reservoir of high-energy excitonic quasiparticles that feeds the exciton-polariton condensate by stimulated scattering^17^. The reservoir interacts with exciton-polaritons repulsively, thus inducing a potential wall^23^,^24^. Above the critical pumping which enables the exciton-polariton condensation, the condensate is created in a multi-mode regime^24^ and therefore occupies multiple energy eigenstates of the billiard. The shape and deformations of the walls enclosing the billiard are precisely controlled by an optical pump^22^, thus enabling us to choose two neighbouring energy states and drive them to degeneracy in the form of a diabolical point. Moreover, this precise control over the billiard shape allows us to encircle the diabolical point along a closed path in a plane of two deformation parameters.

Previously, an exciton-polariton Sinai billiard was used to demonstrate the Berry phase arising from encircling an *exceptional point*^22^. Unlike a Hermitian diabolical point considered here, an exceptional point is a *non-Hermitian* spectral degeneracy, which results in a branch-point singularity in the complex space of the system’s eigenvalues. In order to demonstrate the Berry phase arising from the non-Hermitian nature of the system, one needs to control *imaginary parts* of the eigenvalues (linewidths), as well as the position of the energy levels. As demonstrated in ref. 22, this additional degree of control can be accessed by changing the thickness of the billiard walls. Indeed, since the walls are created by an optical pump, which injects polaritons into the system, the change in thickness affects the overlap between the billiard modes and the gain region, thus effectively changing the Q-factor of the optically induced cavity for coherent exciton-polariton waves. Remarkably, a similar principle of controlling the Q-factor by modifying the geometry of the external cavity walls has also been theoretically proposed for resonant cavities constructed from epsilon-near-zero electromagnetic metamaterials, where it was shown that, at the same time, the position of cavity resonances could remain geometry-invariant^25^.

In contrast to the previous study^22^, here we do not exploit the possibility to independently control the width of the spectral resonances associated with the billiard modes, i.e. we do not manipulate the imaginary parts of the eigenvalues. It is therefore important that the thickness of the billiard wall remains intact while we deform the billiard shape, and therefore we can treat the spectral degeneracy as purely Hermitian.

The transmutations of the eigenstates near the point of degeneracy is reflected in the evolution in their nodal lines. Therefore, in analogy with nanoscopic experiments on surface electrons in quantum corrals^10^, the Berry phase in our experiments is observed by direct imaging of the nodal patterns of the exciton-polariton probability densities, without the need to perform interferometry.

## Results

Classical billiards are two-dimensional areas of arbitrary shape surrounded by hard walls of infinite height, where a classical particle exhibits ballistic motion within elastically reflecting boundaries^26^. Quantum counterparts of classical billiards have become a paradigmatic system for studies of transition from integrable (regular) to chaotic behaviour in quantum regime^27^. Square billiards are completely integrable and exhibit regular classical motion. Consequently, systematic (arising from symmetry) degenerate states are abundant in square quantum billiards^13^,^27^, as given by the energy eigenvalues *E*_*n*,*m*_ = *E*_0_(*n*^2^ + *m*^2^)/2, where *E*_0_ is the ground state energy, and *n*, *m* = 1, 2, 3 … are quantum numbers. For every combination of *n* and *m*, there is a two-fold degeneracy of the eigenstates |*n*, *m*〉. Any deformation of the square breaks this symmetry and lifts the degeneracy thus splitting the energy of the previously degenerate states. Conversely, by using two deformation parameters, accidental degeneracies of energy levels can be found without restoring any reflection or rotational symmetry of the system.

**Figure 1:**
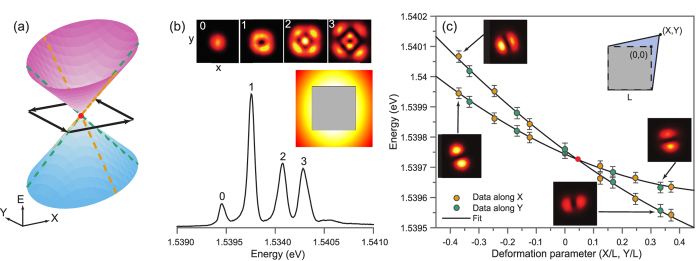
(**a**) The theoretical energy surfaces of the first two excited states denoted by |1〉 and |2〉 of an infinite-well, hard-wall deformed rectangular billiard in the *X* − *Y* parameter space showing the loop (black arrows) used to obtain the Berry phase and the diabolical point (red dot). The energy is normalised with respect to the ground state. (**b**) Typical experimental spectra of exciton-polaritons in an asymmetric square billiard (photoluminescence intensity, a.u.). The numbered peaks correspond to: 0 – a ground state, 1 – the two lowest-order excited states |1〉 and |2〉 at the point of degeneracy, 2 and 3 – higher-order states. Inset: (top) The corresponding real space images of the ground and excited states, and (bottom) the spatial pump profile which creates the billiard (see Methods). (**c**) The experimentally measured energy corresponding to the dashed lines on the surfaces in (**a**). Positions of the individual energy levels are determined from the spectroscopic peaks shown in (**b**). The intersection (red dot) corresponds to the degeneracy point inferred from the polynomial fit (solid curve). The four images shown are the measured real space (*x*, *y*) probability densities of the two eigenstates away from the diabolical point. Inset: The deformation parameters (*X*, *Y*) shown as coordinates of the deformed corner. Negative values correspond to the shift inwards with respect to the square boundary indicated by the dashed contour; this boundary corresponds to the inner area of the billiard shown in the inset panel of (**b**).

The energy surfaces in parameter space of degenerate states can form a conical intersection—a diabolical point, as demonstrated in Fig. 1(a). The energy surfaces shown in Fig. 1(a) are calculated by finding energy eigenvalues in a rectangular billiard (one side longer by 10%) bordered by hard, infinite walls. This reduced symmetry, compared to that of a square, displays no degeneracy for several of the lowest-lying energy eigenstates. One corner of the billiard is then shifted, as shown in the inset of Fig. 1(c), and its coordinates *X*, *Y* are the two deformation parameters used to find an accidental degeneracy.

In the vicinity of a diabolical point the behaviour of the energy surfaces can be modelled by a 2 × 2 Hamiltonian of a generic two-level system given by:


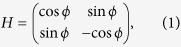


where *X* = *r* cos *ϕ* and *Y* = *r* sin *ϕ*, and *ϕ* is the deformation angle. The eigenvalues of this Hamiltonian, *E*_±_ = ±*r*, form two intersecting circular cones in the *E* − *X* − *Y* space similar to that shown in Fig. 1(a). The eigenvectors are linear combinations of the degenerate eigenstates and are functions of the angle *ϕ* only:


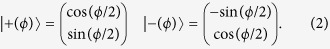


It is easy to show that, if we follow a loop that encloses a diabolical point in the parameter plane, a *π*-phase shift, i.e. the Berry phase, will result, i.e. 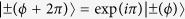. This means that it takes two full rotations around the point of degeneracy to restore the original wavefunction.

In our experiment, we create the quantum billiard for exciton-polariton waves by employing the concept of optically-defined polariton potentials^22^,^23^,^24^,^28^,^29^,^30^,^31^,^32^,^33^ (see Methods for details). These potentials are induced in the quantum well embedded in a microcavity due to the strong Coulomb repulsion between the high-energy excitonic reservoir created by an optical pump and Bose-condensed exciton-polaritons. The spatial distribution of the reservoir particles is defined by the intensity distribution of the optical pump, which, in turn, can be shaped by a spatial light modulator^22^,^24^,^28^,^29^,^31^. Here, we used a digital micromirror device (DMD) to mask a slightly elliptical laser beam profile and create the reservoir density distribution similar to the inset of Fig. 1(b), where the higher pump intensity (depicted by a lighter colour) corresponds to the higher reservoir density. Above a threshold pumping power, exciton-polaritons condense into a macroscopic phase-coherent state. The energy spectra and spatial probability density distribution for the macroscopic wavefunctions of the exciton-polariton condensate are directly imaged and analysed via the microcavity photoluminescence, as described in Methods.

For the pump powers above condensation threshold, we find that the exciton-polariton condensate occupies several energy states of the induced potential, which are represented by well separated lines in the spectrum, as seen in Fig. 1(b). Due to the reduced symmetry of the pumping profile, we found that the first and second excited states, which we denote |1〉 and |2〉, are not degenerate at (0, 0), i.e. when the corner is not shifted, see Fig. 1(c). These states correspond to the degenerate states |1, 2〉 and |2, 1〉 in an ideal square billiard. By shifting the position of the corner, we can drive the two energy levels to become degenerate at a particular point in the *X* − *Y* parameter plane, which is a diabolical point. For the billiard we used, the degeneracy occurs at (*X*/*L*, *Y*/*L*) = (0.05, 0.05) as deduced from the polynomial fit of the experimental data points in Fig. 1(c). To confirm that there is indeed a true degeneracy, a Berry phase of *π* should be observed as a result of a cyclic change of the parameters along a closed planar contour enclosing the diabolical point.

To visualise the Berry phase, we need to follow the transformation of the phase of the eigenstates as we traverse a contour in the plane of the two deformation parameters. Near-field (position-space) imaging after a spectrometer allows us to image the probability density of the eigenstates of a polariton billiard (see Methods). The Berry phase can then be determined without the need for an interference experiment following the protocol initially proposed in refs 10 and 14 for for surface electrons in quantum corrals and classical microwave billiards, respectively. This protocol relies on precise imaging of the nodal lines of the eigenstates associated with the spectral degeneracy, as described in detail below. It is straightforward to implement in our experiment since both the eigenstates involved are two-lobed modes with a well-defined orientation of the nodal line. However, due to the finite energy linewidth, the deformation should be large enough to avoid the overlap between the two modes.

**Figure 2:**
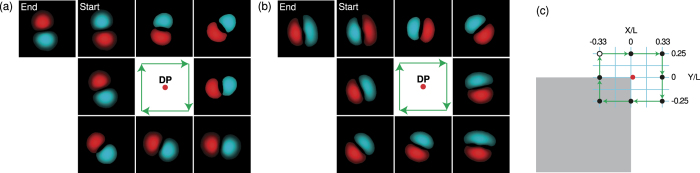
Encircling the diabolical point. Panels (a,b) show the change in the non-dynamical phase of the first (**a**) and second (**b**) eigenstates of the polariton billiard. An initial phase was assigned to each lobe of the wavefunction at the start; colour contrast corresponds to the *π* phase difference. The phase of each successive wavefunction is set in such a way that it exhibits a smooth transition from the preceding point. After one loop, the phase of each state is flipped, and a Berry phase of *π* is accumulated. (**c**) Sequence of the billiard deformations (*X*, *Y*) corresponding to the loops around the diabolical point, as shown in (**a,b**). The red dot marks the point of accidental degeneracy, and the open dot marks the start and the end point of the respective loops.

Figure 2 shows the transformation of the probability density and the phase of the two eigenstates, within the measurement protocol, when a loop enclosing the diabolical point is followed in the parameter plane. An arbitrary starting point in *X* − *Y* plane is chosen and the initial phase is set by defining one lobe of the probability density as positive (zero phase) and the other as negative (*π* phase). Each panel in Fig. 2 therefore shows the experimentally measured probability density distribution multiplied by the corresponding phase, with the *π* phase step across the nodal line. Then we move to the nearest data point clockwise in the *X* − *Y* plane and assign the relative phase in such a way as to ensure a smooth transition from the previous point. The process is repeated for the subsequent data points until a loop enclosing the diabolical point is completed. At the end of the loop around the diabolical point, the phase of each eigenstate has flipped, i.e. accumulated the *π*-phase shift, which corresponds to the geometric Berry phase. A second loop is therefore necessary to return to the original wavefunction.

**Figure 3:**
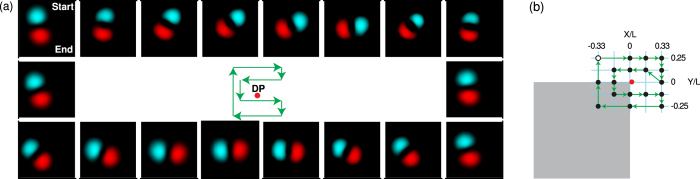
Avoiding the diabolical point. (**a**) The evolution of the phase of the first excited eigenstate in a loop without enclosing the diabolical point in the parameter plane. After a complete loop, there is no phase shift. Inset shows schematics of the path taken in the parameters plane. (**b**) Sequence of the billiard deformations (*X*, *Y*) corresponding to the loop avoiding the diabolical point, as shown in (**a**). The red dot marks the point of accidental degeneracy, and the open dot marks the start and the end point of the loop.

A different loop in the plane of the two deformation parameters can be chosen to *avoid* the diabolical point, as shown in Fig. 3. Following the same procedure, one can see that there is no accumulated phase shift after completing a single loop, which is in contrast to Fig. 2. This suggests that there is indeed a true degeneracy in this two-level system, even though there are uncontrolled asymmetries in the billiard shape arising from imperfections of the optical pumping profile.

**Figure 4:**
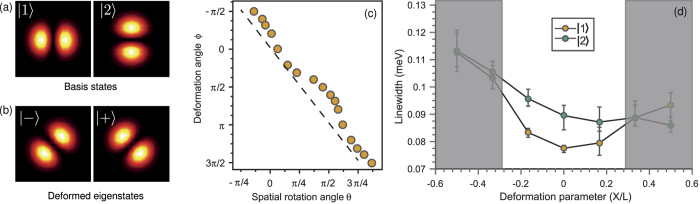
Theoretically calculated probability densities (**a**) of the chosen basis states in Eq. 1 and (**b**) of the mixed states due to deformation angle *ϕ* = *π*/2 showing rotation in real space by *θ* = *π*/4. (**c**) Experimentally measured rotation angle *θ* of the probability densities corresponding to deformed eigenstates as a function of the polar angle *ϕ* of the deformation parameters *X*, *Y*. Dashed line corresponds to the relationship *θ* = *ϕ*/2 expected to arise due to an ideal diabolical cone shown in Fig. 1(a). (**d**) Experimentally measured linewidths of the states |1〉 and |2〉 as a function of a single deformation parameter. Shaded regions represent values of the deformation parameter, for which the simple diabolical point model of the Hermitian spectral degeneracy (Eq. 1) is no longer applicable.

It is well known that perturbation of the billiard potential due to shape deformations causes mixing of the original eigenstates as shown in Eq. 2. Importantly, here this mixing is manifested as a rotation of the eigenstates in real space as observed from the evolution in probability density in Figs 2 and 3. This rotation can be understood with the help of Eq. 2. Indeed, by choosing basis states |1〉 and |2〉 corresponding to the two-lobed modes in Fig. 4(a), the corresponding eigenstates of the deformed billiard |+〉 and |−〉 [Fig. 4(b)] can be viewed as the basis states with the nodal line rotated by the angle *θ* = *ϕ*/2, where *ϕ* is the polar angle in the plane (*X*, *Y*) of the deformation parameters. For an ideal system modelled by Eq. 1, the relationship between the two angles is linear [dashed line in Fig. 4(c)]. The experimentally measured relationship between the deformation angle and the rotation angle of the eigenstates shown in Fig. 4(c) displays a slightly nonlinear behaviour. This is primarily due to the elliptical cone shape of the energy sheets of the two states exploited in this experiment in contrast to the circular cone of the ideal model shown in Fig. 1(a). Nevertheless, it is this simple correspondence between the billiard deformation angle and the angle of rotation of the nodal lines for the two-lobe probability density distributions of the states |1〉 and |2〉 that enables us to observe the Berry phase directly, without the need for interferometry or numerical modelling^22^.

## Discussion

It should be noted that, due to the intrinsic open-dissipative nature of our system, the quantum billiards for exciton-polaritons are non-Hermitian^22^. Nevertheless, as pointed out in the Introduction, here we do not change the thickness of the billiard walls [coloured area in Fig. 1(b), inset] and therefore, in contrast to ref. 22, do not actively manipulate the relative overlap of the different billiard modes with the gain region created by the optical pump. As a consequence, imaginary parts (linewidths) of the two energy eigenvalues used to construct the diabolical point *grow concurrently* and *nearly monotonically* as the parameters *X*, *Y* are detuned from the point of exact degeneracy. This ensures that we are dealing with an equivalent of a Hermitian spectral degeneracy well modelled by Eq. 1 rather than an exceptional point considered in ref. 22. This behaviour of the energy eigenvalues and spectral linewidths in an exciton-polariton billiard is similar to that observed in dielectric optical microcavities^34^,^35^. Indeed, it was established^35^ that, in open resonators (classical wave billiards), the linewidths of two neighbouring energy levels change monotonically as the eigenvalues move along the *diabatic lines* [e.g., dashed lines on the diabolic cone in Fig. 1(a)] away from the point of degeneracy. This is exactly the behaviour observed here as we move further away from the point of degeneracy, as shown in Fig. 4(d). However, for large deformations the change in the linewidths of the states |1〉 and |2〉 is no longer concurrent and monotonic. Therefore, in order for the simple theoretical model Eq. 1 to be applicable, we have to ensure that the values of the deformation parameters remain sufficiently small. Large deformation parameters result in larger deviation of the experimentally observed intersection of energy levels from the exact diabolical cone, which manifests itself in further departure from the linear dependence *θ*(*ϕ*) (Fig. 4(c)).

To summarise, we have visualised the quantum Berry phase by exploiting the existence of a diabolical spectral degeneracy in a deformed square billiard for macroscopic coherent waves of microcavity exciton-polaritons. By constructing a loop in the parameter space that encloses the diabolical point, we observed a *π* geometric Berry phase shift of the eigenmodes. Consequently, no accumulated geometric phase was observed when the closed path avoided the diabolical point. We have also demonstrated that, with a proper choice of the basis, the two eigenstates in the non-separable system are a superposition of the degenerate states which depends on the deformation angle *ϕ* that rotates the original state by an angle of *ϕ*/2. Our experimental observations agree well with a simple two-level Hamiltonian model near a diabolical point.

Our work illustrates that, due to the opportunities presented by direct imaging of the condensate spectra and nodal structure of the spatial probability densities (wavefunctions) via the cavity photoluminescence, exciton-polariton billiards represent an ideal system for visualising and exploring geometric phase effects in quantum systems.

## Methods

In our experiment, exciton-polaritons are created in an AlAs/AlGaAs microcavity containing 12 GaAs quantum wells (~13 nm wide each) sandwiched between distributed Bragg reflector mirrors (32/36 mirror pairs). The quasiparticles are excited by an off-resonant, linearly polarised pump beam derived from a continuous wave (CW) Ti:sapphire laser operating at 732 nm, and driven to condensation above the threshold power of ~0.079 mW/*μ*m^2^ at ~6 K maintained inside a continuous flow microscopy cryostat. Details of the experimental apparatus can be found in ref. 22.

A quantum billiard for exciton-polariton waves is created by spatial shaping of the optical pump using a digital micromirror device (DMD), as detailed in ref. 22. The pump shape reflected by the DMD mirror and re-imaged onto the surface of the sample through a high NA objective lens is shown in Fig. 1(b), inset. The inner length of the square billiard wall is *L* = 12 *μ*m, and the wall thickness is 4.5 *μ*m. Although the mask created by the DMD has a square symmetry, the laser profile is slightly elliptical thus reducing the spatial symmetry of the pump to that of a rectangle [see inset of Fig. 1(b)]. Cavity photoluminescence resulting from decay of exciton-polaritons and release of coherent photons is collected via the same objective lens and analysed using a spectrometer and a camera. Analysis of the microcavity emission therefore delivers the spectrum of exciton-polaritons [as seen in Fig. 1(b,c)] and real-space images of the spatial probability distribution for the exciton-polariton condensate, as seen in Figs 1, 2 and 3. Above the threshold pump power, exciton-polaritons occupy multiple eigenstates of the optically defined trap^22^,^24^,^28^,^29^,^30^,^32^ in the shape of the rectangular potential, which are resolved by analysing the spectrum of the photoluminescence with the aid of energy tomography. Here, the tomography refers to the process of the real-space imaging of the condensate probability density by capturing the near-field emission after it passes through the spectrometer. This allows us to select and analyse the spatial distribution of the probability density corresponding to a particular wavelength (energy), rather than imaging an integrated emission in a wide spectral range.

From the tomography, we obtain the spectra as a function of real space coordinates (*x*, *y*). Despite the large linewidths, the peak for one mode is easily isolated by choosing regions in real space where the probability density of the other mode is minimal. The spectrum at each pixel in that region is then fitted with a Gaussian function without any free parameters. The centre and the full width at half maximum of the Gaussian fit correspond to the energy and linewidth of the mode, respectively. The standard deviation of the fit parameters corresponds to the error bars in Figs 1(c) and 4(d). The numerical error from each individual fit is an order of magnitude smaller.

The deformation of the billiard potential is achieved by reprogramming the DMD to reflect a different shape. In particular, the loops in the parameter plane displayed in Figs 2(a,b) and 3(a) are produced by the deformation sequences depicted in Figs 2(c) and 3(b), respectively. Within this range of deformations, the widths of the spectral lines associated with the eigenstates |1〉 and |2〉 change simultaneously and nearly monotonically as the system is deformed away from the degeneracy point, as seen in Fig. 4(d).

## Additional Information

**How to cite this article**: Estrecho, E. *et al*. Visualising Berry phase and diabolical points in a quantum exciton-polariton billiard. *Sci. Rep.* **6**, 37653; doi: 10.1038/srep37653 (2016).

**Publisher's note:** Springer Nature remains neutral with regard to jurisdictional claims in published maps and institutional affiliations.
